# Growth Hormone Therapy for Small for Gestational Age Short Stature Develops Type 2 Diabetes

**DOI:** 10.1155/2023/9912817

**Published:** 2023-11-15

**Authors:** Naohiro Nomura, Yuko Tanabe, Miki Minami, Junji Takaya, Kazunari Kaneko

**Affiliations:** ^1^Department of Pediatrics, Kansai Medical University, Osaka, Japan; ^2^Department of Pediatrics, Kawachi General Hospital, Osaka, Japan

## Abstract

Growth Hormone therapy has been shown to induce transient insulin resistance in children, and there is concern regarding the diabetogenic potential of GH therapy in children born small for gestational age (SGA). In this case, female patient born SGA with a weight of 2,750 g (−1.73 standard deviation (SD)) and length of 45.5 cm (−2.6 SD). The patient's father and paternal grandfather were diagnosed with type 2 diabetes mellitus. At 3 years of age, the patient presented with short stature; height and weight were 85 cm (−2.5 SD) and 13 kg (−0.19 SD), respectively. She was placed on GH therapy. At 11 years of age, her fasting blood glucose and hemoglobin A1c levels were 116 mg/dL and 7.4%, respectively. Blood test results were negative for anti-glutamic acid decarboxylase and anti-islet antigen-2 antibodies. The patient discontinued GH therapy and started diet therapy and oral metformin (500 mg/day) administration. Five months later, the hemoglobin A1c level was 5.3% and glycemic control further improved. To our knowledge, family history may be an important risk factor for GH-induced diabetes. So, the GH dosage for patients born SGA with family history of diabetes should be adjusted so as not to be too excessive, and long-term follow-up studies will be required to evaluate fully the effects of GH therapy for them.

## 1. Introduction

Children born small for gestational age (SGA) are defined as infants born with a weight and/or height that is two standard deviations (SDs) below the mean for their gestational age. Although the great majority of children born SGA catch up to their peers in the first years of life, up to 15% do not catch up by 2 years of age, and many of these remain abnormally short. These children are candidates for growth hormone (GH) treatment to improve their height outcomes [1].

Small for gestational age affects glucose metabolism and insulin sensitivity [2], which entails the subsequent risk of developing type 2 diabetes mellitus (DM2) [3]. GH therapy has been shown to induce transient insulin resistance in children, and there is concern regarding the diabetogenic potential of GH therapy in children born SGA [4].

## 2. Case Presentation

**Figure 1:**
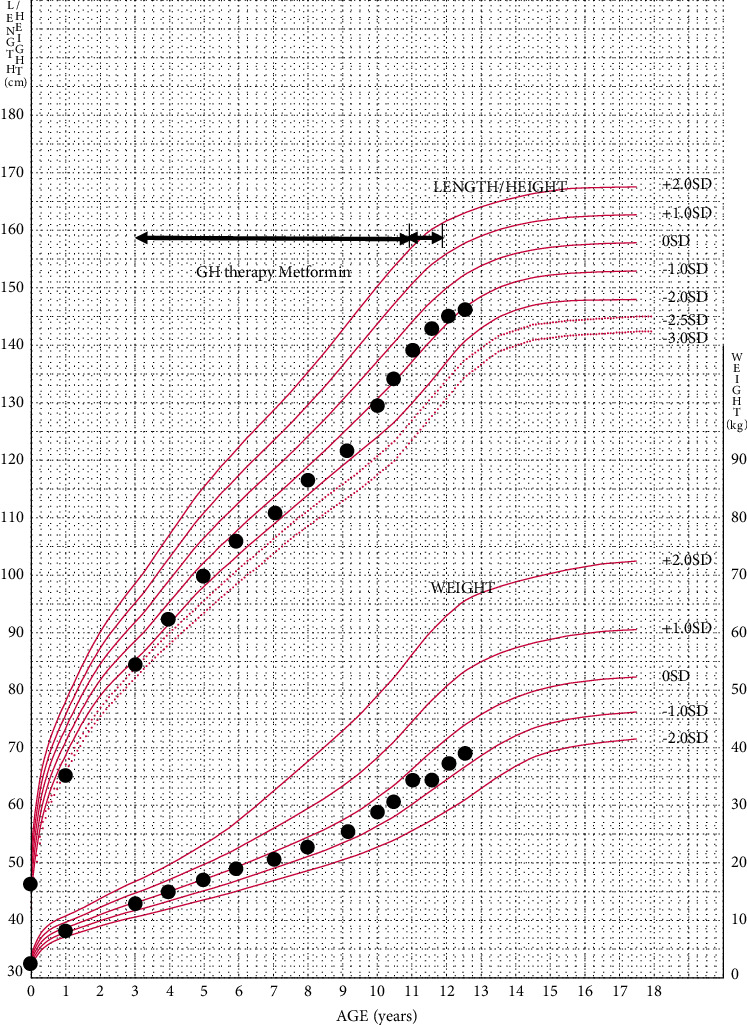
Growth chart and clinical events of the patient. GH treatment was started at 3 years of age for SGA short stature. Her statural response to GH therapy was good. At 11 years of age, she was diagnosed with type 2 diabetes mellitus, completed GH treatment, and started oral metformin.

The patient was born at 41 weeks and 4 days of gestational age with a weight of 2,750 g (−1.73 standard deviation (SD)) and length of 45.5 cm (−2.6 SD). The patient's father and paternal grandfather were diagnosed with DM2. At 3 years of age, the patient presented to us with short stature and a height and weight of 85 cm (−2.5 SD) and 13 kg (−0.19 SD), respectively. She was placed on GH therapy for children who are born SGA and manifest persistent short stature. The GH dosage was increased from 0.23 to 0.35 mg/kg/week among first two years and then was adjusted for weight gain. Her statural response to GH therapy was good. Her blood hemoglobin A1c (HbA1c) level was not affected by the increase in the GH dosage. HbA1c was measured using the high-performance liquid chromatography method. The patient's height and body weight growth curves are shown in Figure 1.

During the GH treatment, serum insulin-like growth factor-1 values remained around +1 SD for her age (Table 1). At 9 years and 5 months of age, she exhibited breast development. With pubertal development, her HbA1c level was slightly increased, though it remained 5.5%–6.0% in childhood.

At 11 years of age, the patient had a height of 139.2 cm (−0.67 SD), weight of 34.1 kg (+4.6%), and body mass index of 17.6 kg/m^2^ (+0.56 SD). She had Tanner III breast and pubic hair development and otherwise an unremarkable physical examination. Her fasting blood glucose and HbA1c levels were 116 mg/dL and 7.4%, respectively. Serum liver enzyme levels and renal function levels were within normal ranges. Blood results were negative for anti-glutamic acid decarboxylase and anti-islet antigen-2 antibodies. On the basis of these results, the patient was diagnosed with DM2.

Soon after diagnosis, the patient discontinued GH therapy and started diet therapy and oral metformin administration (500 mg/day) at 11 years and 3 months of age. The blood HbA1c level further increased to 8.5% at 11 years and 4 months of age (Table 1). Two months after starting the treatments, her HbA1c levels improved to 6.9%. Five months later, the patient's HbA1c level was 5.3% and her glycemic control further improved. Since then, improvements in glucose intolerance and hyperinsulinemia have been observed, and oral administration of metformin was discontinued at 10 months. Currently, no recurrence of impaired glucose tolerance has been observed in the patient, who is being followed up without resuming GH treatment.

## 3. Discussion

Previous papers have reported that GH treatment in SGA children may not increase the risk of DM2 or metabolic syndrome [6, 7]. However, long-term follow-up studies will be required to determine whether GH therapy increases the risk of DM2 in subjects born SGA. The increased insulin resistance in the current patient may be explained as follows. (1) SGA/low birth weight is considered a risk factor for developing glucose intolerance. (2) Term SGA infants like the present case have a higher risk of developing noncommunicable diseases than preterm SGA infants [8]. (3) During puberty, the growth-promoting period, increased GH and sex hormone secretion may elevate the risk of insulin resistance. (4) This patient had a family history of diabetes spanning three generations. (5) Long-term GH treatment may aggravate insulin resistance. Several studies on children have shown cutoff values of insulin resistance between 2.5 and 3.2, according to HOMA-IR [9, 10]. Circulating HbA1c levels were within the normal range in all patients, urine sugar was negative, and no diabetes was observed.

## 4. Conclusion

The potential impact of GH on DM2 risk is encouraging in that short-term effects of GH treatment upon fasting glucose and insulin resistance did not result in an increased risk for DM2. However, family history may be a more important risk factor. So, the GH dosage for patients born SGA with family history of diabetes should be adjusted so as not to be too excessive, and long-term follow-up studies will be required to evaluate fully the effects of GH therapy for them.

## Figures and Tables

**Table 1:** Changes in blood hemoglobin A1c, insulin-like growth factor-1, and hormone levels during treatment with growth hormone.

Age (y/m)	9/6	10/0	10/6	11/0	11/4
HbA1c (%)	5.5	5.8	5.8	7.4	8.5
IGF-1 (ng/mL)	376	366	559	693	542
[M ± SD] [5]	[264 + 0.6 SD]	[302 + 0.2 SD]	[302 + 1.4 SD]	[333 + 1.7 SD]	[333 + 1.0 SD]
LH (mIU/mL)	0.11	0.26	3.45	4.37	4.8
FSH (mIU/mL)	2.39	3.99	4.62	5.57	5.97
Estradiol (pg/mL)	<10	13	15	36	65

HbA1c: glycosylated hemoglobin, IGF-1: insulin-like growth factor-1, M ± SD: median ± standard deviation, LH: luteinizing hormone, FSH: follicle-stimulating hormone, and y/m: years and months of age.

## Data Availability

No data were used to support this study.
